# rCBV- and ADC-based Grading of Meningiomas With Glimpse Into Emerging Molecular Diagnostics

**DOI:** 10.32598/bcn.9.6.417

**Published:** 2018-11-01

**Authors:** Seema Rohilla, Harender K. Garg, Ishwar Singh, Rohtas K Yadav, Dhara B. Dhaulakhandi

**Affiliations:** 1. Department of Radiodiagnosis & Imaging, Post Graduate Institute of Medical Sciences, Sharma University of Health Sciences, Rohtak, Haryana, India.; 2. Department of Neurosurgery, Post Graduate Institute of Medical Sciences, Sharma University of Health Sciences, Rohtak, Haryana, India.; 3. Department of Biotechnology & Molecular Medicine, Post Graduate Institute of Medical Sciences, Regional Cancer Centre, Sharma University of Health Sciences, Rohtak, Haryana, India.

**Keywords:** Meningioma grading, Relative Cerebral Blood Volume, Apparent diffusion coefficient, Genomic, Proteomics

## Abstract

**Introduction:**

This study was conducted to grade meningiomas based on relative Cerebral Blood Volume (rCBV) and Apparent Diffusion Coefficient (ADC) to help surgeons plan the approach and extent of operation as well as decide on the need of any adjuvant radio/chemo therapy. The current and evolving genomic, proteomic, and spectroscopic technologies are also discussed which can supplement the current radiologic methods and procedures in grading meningiomas.

**Methods:**

A total of 35 patients with meningioma prospectively underwent basic MR sequences (T1W, T2W, T2W/FLAIR) in axial, sagittal and coronal planes followed by Diffusion Weighted (DW) imaging having b value of 1000 (minimum ADC values used for analysis). Then, gadobenate dimeglumine/meglumine gadoterate was administered (0.1 mmol/kg at a rate of 4 mL/s) followed by saline flush (20 mL at a rate of 4 mL/s). Next, T_2_*W/FFE dynamic images were acquired; dynamics showing maximum fall in intensity was used for creating rCBV and relative Cerebral Blood Flow (rCBF) maps and calculating rCBV.

**Results:**

Both maximum rCBV and minimum ADC within the tumor were not significant for differentiating benign from malignant meningiomas. A cut-off maximum rCBV of 2.5 mL/100 g in peritumoral edema was 75% sensitive, 84.6% specific, and 83.3% accurate in differentiating benign from malignant meningiomas.

**Conclusion:**

Benign and malignant meningiomas can be differentiated based on maximum rCBV in peritumoral edema but ADC values within the tumor are insignificant in differentiating benign and malignant tumors. rCBV values within tumor, however, may be helpful in subtyping meningiomas, especially transitional and meningothelial meningiomas.

## Highlights

- This study was conducted to grade meningiomas based on relative Cerebral Blood Volume (rCBV) and Apparent Diffusion Coefficient (ADC).
- The results could help surgeons plan the approach and extent of operation as well as decide on the need of any adjuvant radio/chemo therapy.
- Maximum rCBV and minimum ADC within the tumor were not significant for differentiating benign from malignant meningiomas.
- Benign and malignant meningiomas can be differentiated based on maximum rCBV in peritumoral edema.
- ADC values within the tumor are insignificant in differentiating benign and malignant tumors.
- rCBV values within tumor, however, may be helpful in sub-typing meningiomas, especially transitional and meningothelial meningiomas.

## Plain Language Summary

This study was conducted to grade meningiomas based on relative Cerebral Blood Volume (rCBV) and Apparent Diffusion Coefficient (ADC). The current and evolving genomic, proteomic, and spectroscopic technologies are also discussed which can supplement the current radiologic methods and procedures in grading meningiomas. In this regard, a total of 35 patients with meningioma prospectively underwent basic MR sequences (T1W, T2W, T2W/FLAIR) in axial, sagittal and coronal planes followed by Diffusion Weighted (DW) imaging. Then, gadobenate dimeglumine/meglumine gadoterate was administered followed by saline flush. Finally, T_2_*W/FFE dynamic images were acquired; dynamics show maximum fall in intensity used for creating rCBV and relative Cerebral Blood Flow (rCBF) maps and calculating rCBV. Based on the results, maximum rCBV and minimum ADC within the tumor were not able to differentiate benign from malignant meningiomas. Benign and malignant meningiomas can be differentiated based on maximum rCBV in peritumoral edema but ADC values within the tumor are invalid in differentiating benign and malignant tumors. rCBV values within tumor, however, may be helpful in sub-typing meningiomas, especially transitional and meningothelial meningiomas.

## 1. Introduction

Meningiomas account for 15% to 20% of all primary brain tumors and are the most common primary brain tumors after glial tumors (Buetow, Buetow, & Smirniotopoulos, 1991; Gangadhar, Santhosh, & Fatterpekar, 2013; Russell & Rubinstein, 1989). Atypical features such as cystic and necrotic areas, ring-like enhancement, and parenchymal invasion are observed in about 15% of meningiomas and resemble gliomas or metastases. It results in to incorrect radiological reports and wrong treatment strategies (Hakyemez et al., 2006; Hartying, Hartmann, Bonsanto, Sommer, & Sartor, 2004; Qi et al., 2008). Diffusion Weighted Imaging (DWI) shows tumor cellularity, Magnetic Resonance Spectroscopy (MRS) shows metabolism, Diffusion Tensor Imaging (DTI) helps to identify invasion of white matter tracts while MR perfusion shows neocapillary density and permeability, and thus help in tumor grading (Young, 2007).

The latest World Health Organization (WHO) grading of meningiomas shows three categories in this respect: grade I refers to lesions with low proliferative potential and mostly cured only by surgical resection, grade II tumors show infiltration and often show recurrence despite low proliferative activity, and grade III lesions show evidence of malignancy such as cellular atypia and brisk mitotic activity. Meningiomas and hemangioblastomas are considered grade I, atypical meningiomas and hemangiopericytomas grade II, and anaplastic/malignant meningioma and anaplastic hemangiopericytoma grade III (Louis et al., 2007).

It is critical for the clinicians to know grading, response of tumors to therapies such as gamma knife, tumor progression, and regression or recurrence as early as possible to take proper treatment decisions. Diffusion-Weighted Imaging (DWI), Perfusion-Weighted Imaging (PWI), and MRS provide this information earlier than conventional MRI (Gao, Zhang, Zhang, Yu, & Xu, 2011).

## 2. Methods

Thirty-five patients with meningioma were graded based on MR diffusion and perfusion weighted imaging. T1W (TE-15 ms, TR-596 ms, Field of View (FOV)-230 mm, matrix size-(186×256), flip angle-69° and NSA-1), T2W (TE-100 ms, TR-4431 ms, FOV-230 mm, matrix size-(236×512), flip angle-90° and NSA-2) and T2W/FLAIR (TE-120 ms, TR-6000 ms, FOV-230 mm, matrix size-(172×256), flip angle-100°, TI (time to inversion)=2000 ms and NSA-1) sequences were done in axial, sagittal and coronal planes as per requirement on Gyroscan Intera Nova gradient 1.5-Tesla (Philips Imaging system, Best, Netherlands), using a SENSE head coil (six channel phased array coil).

DWI was performed using single shot Echo Planar Imaging (EPI) sequence with a TE-89 ms, TR-2609 ms, FOV 230 mm, matrix size-89×256, flip angle-90° and NSA-3, with a b-value of 1000. ADC maps were created by automated software on workstation (view forum version 5.1) and minimum ADC values were analyzed.

For PWI, the patients were given gadobenate dimeglumine/meglumine gadoterate (0.1 mM/kg at a rate of 4 mL/s) after which 20 mL normal saline was flushed at a rate of 4 mL/s using pressure injector (Medrad® Spectris Solaris® version 008.001-sa). The images were acquired using T_2_*W/FFE dynamic images (TE-30 ms, TR-627 ms, FOV-230 mm, matrix size-128×128, flip angle-40°, and NSA-2) to track first pass of contrast bolus through the area of interest and the dynamic showing maximum fall in intensity was used to create rCBV and rCBF maps and calculating rCBV using automated software in workstation View Forum 5.1.

Statistical analysis included two-sample Wilcoxon rank-sum (the Mann-Whitney) test, the Kruskal-Wallis equality-of-populations rank test, ROC (receiver operating characteristic) curve analysis done in Stata software version 11.2 (Stata Corp LLC, Texas, USA). Final diagnosis was made by histopathology.

## 3. Results

**Figure 1:**
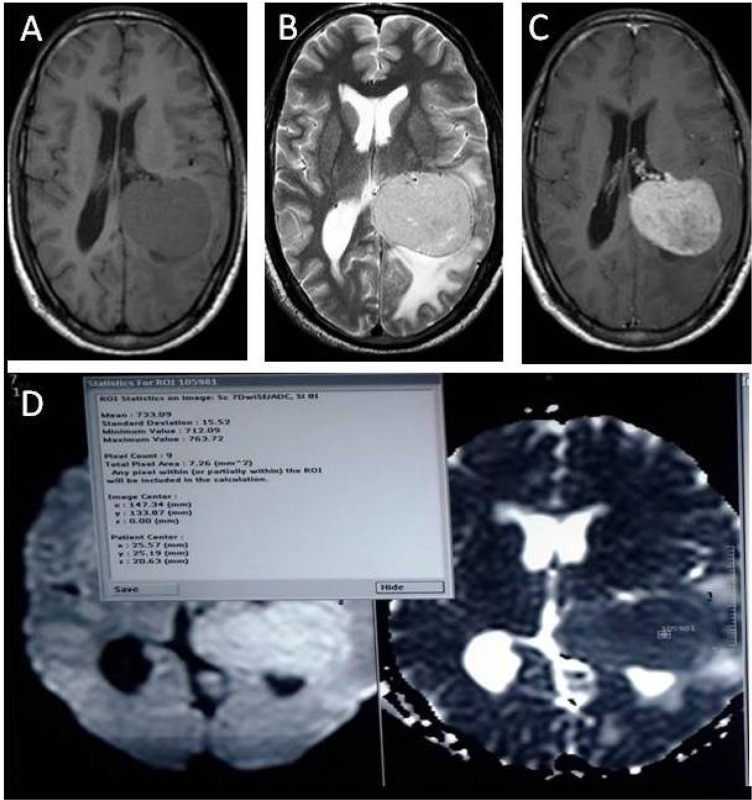

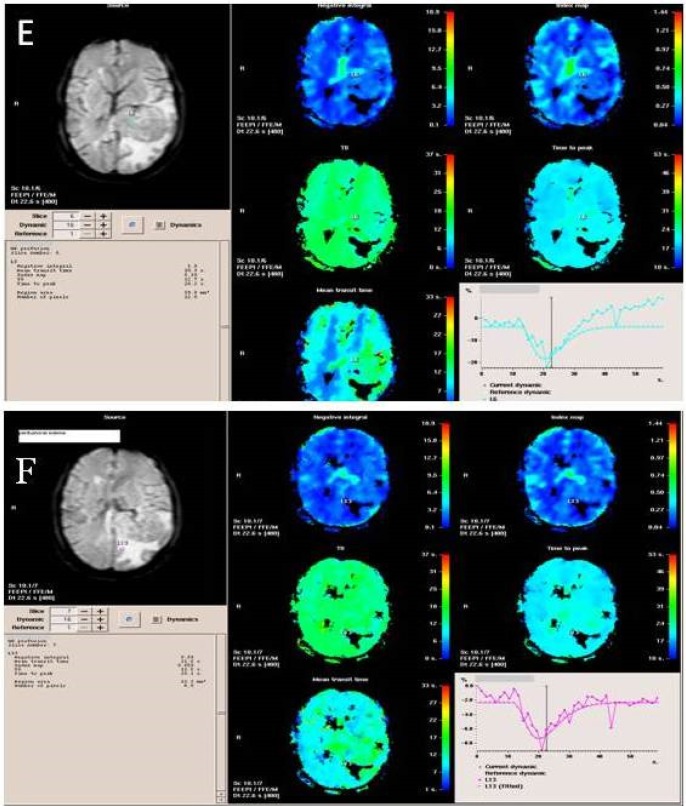
A 40-year-old male with left intraventricular meningioma (benign) A. Axial T1W image showing well defined hypointense lesion in atrium of left lateral ventricle B. T2W image showing hyperintense lesion with peripheral edema C. On contrast enhanced axial T1W images lesion showing homogenous intense enhancement. D. Axial DWI and corrosponding ADC map showing diffusion restriction with minimum ADC value of being 0.733×10^−3^ mm^2^/_s_ E. T2W* first pass perfusion images showing colour coded rCBV maps along with time signal intensity curve and various parameters with rCBV(negative integral) being 1.9 mL/100 g within the tumor F. T2W* first pass perfusion images showing colour-coded rCBV maps along with time signal intensity curve and various parameters with rCBV being 0.69 mL/100 g in peritumoral edema

**Figure 2:**
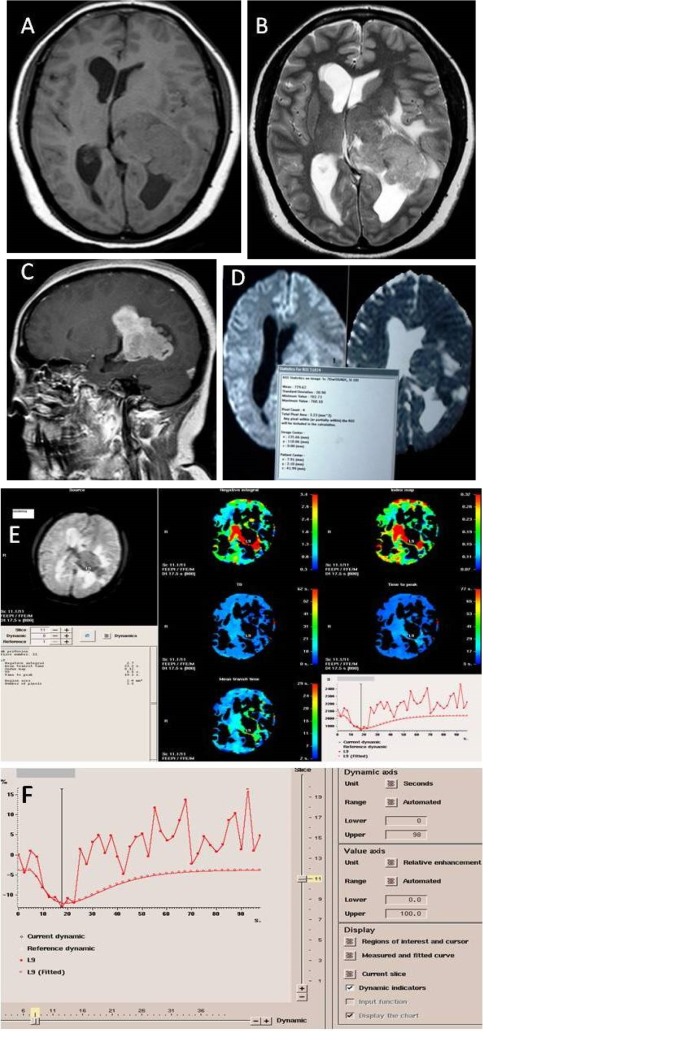
A 40-year-old female with left lateral ventricle meningioma (malignant) A. Axial T1W image showing hypointense lesion arising from left lateral ventricle B. T2W image showing iso- to hyper-intense lesion with perilesional edema causing expansion of left lateral ventricle C. On contrast enhanced saggital T1W image the lesion shows homogenous intense enhancement D. Axial DWI and corrosponding ADC map showing diffusion restriction in its solid part with minimum ADC value being 0.729×10^−3^ mm^2^/_s_ E. T2W* first pass perfusion images showing colour coded rCBV maps along with various parameters with rCBV (negative integral) in peitumoral edema being 2.7 mL/100 g F. Time signal intensity curve in peritumoral edema showing maximum signal intensity drop of 15%

The patients’ age range was 21–75 years. There were 23 females and 12 males. A total of 31 patients had grade I, two grade II, and two grade III meningiomas. Since there were only two cases in grade II and two cases in grade III, the data were not sufficient for statistical analysis. Hence grade II (atypical) and grade III (malignant) were pooled together as atypical/malignant group for proper statistical analysis. Appearance of meningiomas on basic MR sequences is shown in Figures 1 and 2.

### 3.1. DWI

Tumor areas with diffusion restriction appeared hyper-intense on DW images and hypointense on ADC maps. Two-sample Wilcoxon rank sum test for ADC values within the tumor showed the probability of 0.53, implying that its insignificance to grade meningiomas.

### 3.2. PWI

#### 3.2.1. The rCBV within tumor

Two-sample Wilcoxon rank-sum test for rCBV values within the tumor showed the probability of 0.22; indicating that intratumoral rCBV was not significant to grade meningiomas.

#### 3.2.2. The rCBV in Peritumoral edema

**Figure 3:**
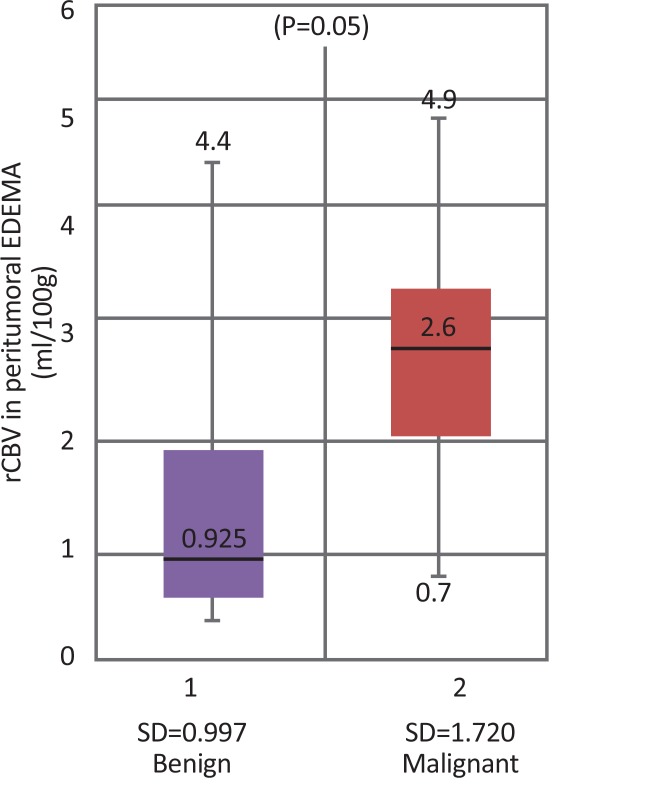
Boxplot diagram showing rCBV values in peritumoral edema in benign vs. malignant meningiomas

Peritumoral edema was observed in 26 cases of grade I meningiomas and all four atypical/malignant meningiomas. Figure 3 shows a box plot showing distribution of peritumoral rCBV values with respect to grades. There was not much overlap in rCBV within the peritumoral edema values of benign and malignant tumors. Therefore, this parameter was helpful to distinguish benign and malignant meningiomas.

**Figure 4:**
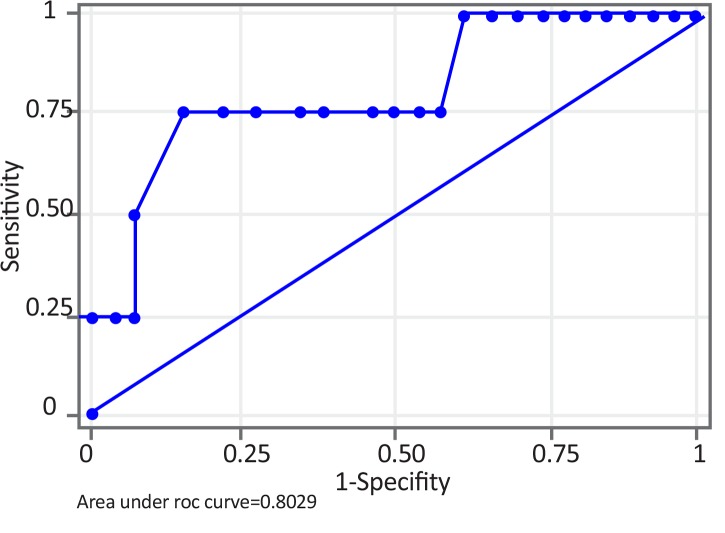
ROC curve of rCBV in peritumoral edema for benign vs. malignant meningiomas A cut-off value of 2.5 mL/100 g for differentiating benign vs. malignant meningiomas gave sensitivity of 75% and specificity of 84.6%. Area Under the Curve (AUC) was 0.8029.

Two-sample Wilcoxon rank-sum test for rCBV values in peritumoral edema showed the probability of 0.05, implying that rCBV in peritumoral edema was significant to grade meningiomas. ROC analysis of perfusion data in peritumoral edema was performed. A cut-off point of 2.5 mL/100 g to differentiate benign vs. malignant meningiomas showed the sensitivity of 75%, specificity of 84.6% and accuracy of 83.3%. Area Under the Curve (AUC) was 0.8029 and standard error 0.1413 with 95% Confidence Interval (CI) of 0.525–1.000 (Figure 4). The range of rCBV values in peritumoral edema in benign and malignant meningiomas is presented in Table 1.

**Table 1:** The rCBV values and percentage of signal drops in Peritumoral edema

**Tumor Grade**	**rCBV Value in Peritumoral Edema (mL/100 g)**	**Percentage Drop in Peritumoral Edema**
Benign	<2.5	<16%
Malignant	≥2.5	≥16%

#### 3.2.3. Percentage drop in Peritumoral edema

**Figure 5:**
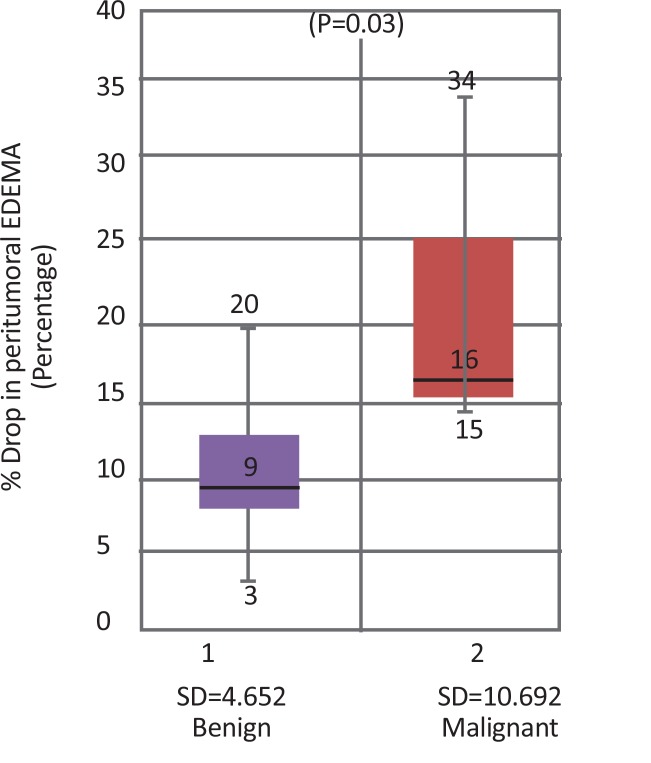
Boxplot diagram showing percentage drop values in peritumoral edema in benign vs. malignant meningiomas

Significance of percentage drop in peritumoral edema was also studied in 27 cases by two-sample Wilcoxon rank-sum test and the probability was 0.0328. Since the value was less than 0.05, hence percentage drop in peritumoral edema was also significantly correlated with tumor grade. The boxplot showing distribution of percentage drop values in peritumoral edema with respect to grades is shown in Figure 5.

**Figure 6:**
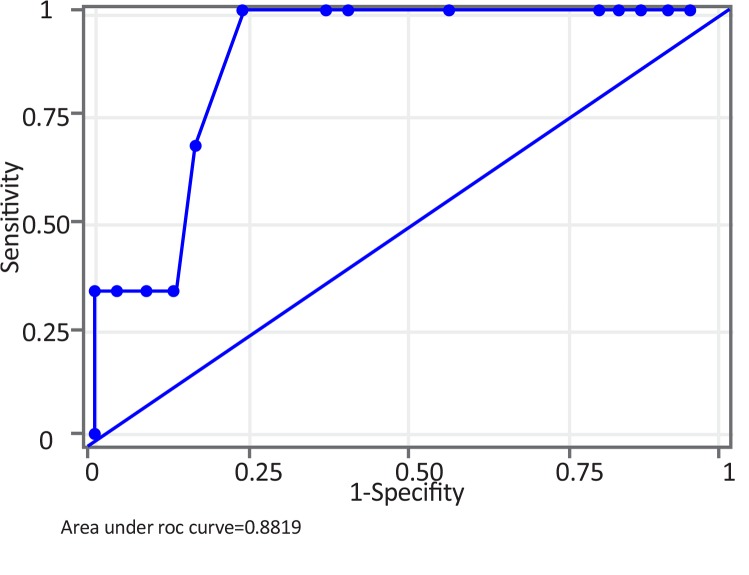
ROC curve of percentage drop in peritumoral edema for benign vs. malignant meningiomas A cut-off value of 16% could differentiate benign from malignant meningiomas with sensitivity of 66.67% and specificity of 83.33%. AUC was 0.8819. (Page 7)

The percentage drop values in peritumoral edema of benign and malignant tumors did not show much overlap. Therefore, this parameter also helps to distinguish benign from malignant meningiomas. On ROC curve analysis, a cut-off point of 16% could differentiate benign from malignant meningiomas with the sensitivity of 66.67%, specificity of 83.33% and accuracy of 81.48%. AUC was 0.8819, standard error 0.0786 with 95%CI:0.727–1.000 (Figure 6). The range of percentage drop values in peritumoral edema with respect to benign and malignant meningioma is presented in Table 1.

The curve for percentage drop was more towards the top and left as compared to the curve for rCBV. AUC for ROC curve of percentage drop was 0.8819 while that of rCBV was 0.8029. Both of these imply that percentage drop in peritumoral edema was a stronger parameter than rCBV in peritumoral edema (though both were statistically significant) in grading meningiomas. Though both of the parameters were based on DSCI (Dynamic Susceptibility Contrast Imaging), the difference may be due to different sample sizes analyzed for the two (n=30 for rCBV while n=27 for percentage drop). But the observation needs further analysis with a larger study.

Out of the total 31 benign meningiomas, 16 were transitional, 12 meningothelial, while only three were fibrous. ADC and rCBV values of benign meningiomas were analyzed according to their subtypes. Only rCBV in tumor was significant for further subtyping of benign meningiomas (P=0.02). The summary statistics of rCBV in the tumor for transitional and meningothelial meningiomas is presented in Table 2. Since the number of cases of fibrous subtype were only three and other subtypes such as angiomatous were not there, their statistical impact was either very low or absent. Hence, in the current study, intratumoral rCBV showed its role in subtyping only transitional from meningothelial meningiomas. This may also be possible for other subtypes, but larger studies are needed to prove that.

**Table 2:** Statistical analysis of rCBV values in different subtypes of tumor

**Tumor Subtype**	**Mean (mL/100 g)**	**SD**	**Range (mL/100 g)**
Transitional meningiomas	3.1	2.276	1.2–8.4
Meningothelial meningiomas	7.8	5.626	0.99–20.7

## 4. Discussion

Meningiomas are most common extra-axial tumors mainly observed in the sixth and seventh decades of life. It is important to segregate typical and atypical meningiomas for proper treatment decisions (Demir, Iplikcioglu, Dincer, Arslan, & Sav, 2006). Increased angiogenesis is correlated with higher rCBV. Increased viscosity, cellular density, and lower extracellular space reduce ADC. Thus, tumor grading is directly proportional to rCBV and inversely proportional to ADC values.

### 4.1. DWI investigation

Only a few studies evaluated the role of DWI to grade meningiomas. Although some studies show that atypical/malignant meningiomas have significantly lower ADC than benign meningiomas, other studies suggest that the difference is not statistically significant. According to Filippi et al. (2006), ADC values are lower in malignant meningiomas than normal brain and are higher in benign meningiomas than normal brain and that the ADC values of malignant and benign meningiomas differ significantly (P<0.00029).

Nagar et al. (2008) also found that atypical/malignant meningiomas had significantly lower mean ADC than benign meningiomas (P<0.0001). Mean±SD normalized ADC ratio of the atypical/malignant group (0.91±0.18) was also significantly lower than that of the benign group (1.28±0.11; P <0.0001) and there was no overlap between the groups. Yin et al. (2012) also reported significant difference between the Mean±SD ADC values of typical and atypical/malignant meningiomas (0.97±0.21×10^−3^ vs. 0.85±0.17×10^−3^ mm^2^/s). The Mean±SD normalized ADC ratios were lower in the atypical/malignant group (1.09±0.23) than in the benign group (1.24±0.25) and the difference was statistically significant (P=0.002).

In the current study ADC values were not significant to differentiate benign from atypical/malignant meningiomas as was evident from the probability of 0.53. Santelli et al. (2010) also reported that the difference between Mean±SD ADC of typical and atypical/malignant meningiomas (0.964±0.192×10^−3^ vs. 0.923±0.085×10^−3^ cm^2^/s, P=0.3 for Student t test) or ADC ratio (1.266±0.290 vs. 1.185±0.115, P=0.2 for Student t test) respectively was not significant. Neither ADC values nor ADC ratios were significant to subtype meningiomas though a rather significant difference was observed between meningothelial and transitional meningiomas (post hoc analysis P=0.06).

According to Stadnik et al. (2001), diffusion-weighted echo-planar imaging do not help to evaluate tumor extension. They also found that contrast was generally lower on diffusion-weighted images and ADC maps against conventional MR imaging. Sanverdi et al. (2012) retrospectively evaluated conventional MR and DW images of 177 adult patients with meningiomas and found that the Mean±SD ADC values and ratios of benign meningiomas were 0.99±0.12×10^−3^ mm^2^/s and 1.22±0.07, respectively. ADC Mean±SD values for atypical and malignant groups were both 0.84±0.1×10^−3^ mm^2^/s. The ADC Mean±SD ratios were 1.05±0.1 and 0.96±0.2 for atypical and malignant meningiomas, respectively.

The mean ADC ratios did not differ significantly among the three subtypes (ANOVA; P≥0.05). Therefore, DW MR imaging do not add to grade or subtype meningiomas. Ignjatovic et al. (2014) found that ADC values were not significantly different between meningothelial, fibroblastic, and cystic meningiomas. The current study could not subtype meningiomas on the basis of ADC values.

### 4.2. PWI investigation

All meningiomas were ‘hot’ (red, orange, and yellow) on rCBV color maps as they had higher blood volume than surrounding tissue (including peritumoral edema). There was no clear interface between peritumoral edema and the surrounding brain tissue. The peritumoral edema around malignant meningiomas was slightly hotter or be the same colors (green and blue) as normal white matter (blue). On the other hand, both tumor and peritumoral edema of benign meningiomas had colors similar to that of normal brain tissue. The rMTE color maps do not show clear interface between tumor and peritumoral edema of meningiomas.

The current study could not differentiate benign from atypical/malignant meningiomas on the basis of intratumoral rCBV since P value was 0.22, well above 0.05. The rCBV in peritumoral edema was, however, significant enough to grade meningiomas as P value was 0.05. Zhang, Rödiger, Shen, Miao and Oudkerk (2008) found that the rCBV values in the parenchyma were not significant (P>0.05), but those in the peritumoral edema were significant (P<0.05) to differentiate benign from malignant meningiomas. Comparison between the study by Zhang et al. (2008) and the current study is presented in Table 3. Zhu, Zhou, Wang, Gao and Qi (2002), however, found that the mean rCBV ratios between grade I, and grades II and III were 8.84 and 3.23, respectively, which were significantly different. They concluded that the average rCBV of meningiomas were significantly higher than that of the normal brain tissue and correlated well with vascularization, which is one of the criteria for histological analysis.

**Table 3:** Comparative rCBV values to differentiate benign and malignant meningiomas

**Study**	**Benign Meningioma**		**Malignant Meningioma**	

	**rCBV in Tumor (mL/100 g)**	**rCBV in Peritumoral Edema (mL/100 g)**	**rCBV in Tumor (mL/100 g)**	**rCBV in Peritumoral Edema (mL/100 g)**
Zhang et al. (2008)	7.16±4.08	1.05±0.96	5.89±3.86	3.82±1.39
Our study	5.51±4.59	1.32±0.99	7.95±4.34	2.7±1.72

Values are presented as Mean±SD.

The current study as well as the study by Zhang et al. (2008) revealed that rCBV ratios in periphery of malignant meningiomas were higher than those of the periphery of benign meningiomas, which may be due to tumor invasion and angiogenesis in the surrounding brain tissue (Arai, Kashihara, & Kaizaki, 2006). However, intratumoral rCBV measurements do not differ significantly among benign and malignant meningiomas, which can be due to hypervascularity of all grades of meningiomas.

### 4.3. Percentage drop

The current study also investigated the role of other parameters such as percentage drop, percentage signal recovery in tumor as well as peritumoral edema, and the slope of signal recovery curve in tumor and peritumoral edema. Out of these, only the percentage drop in peritumoral edema resulted in P value of 0.03 and hence significantly correlated with tumor grade.

The current study data were not evenly distributed for subtyping of meningiomas as there were only three cases of fibrous type and there was no case of angiomatous meningioma. The rCBV value in tumor was significant to subtype meningiomas (mainly transitional and meningothelial) (P=0.02). Santelli et al. (2010) showed that ADC values or ADC ratios were not significant to sub-type meningiomas, although there was a rather significant difference between meningothelial and transitional subtypes (post hoc analysis P=0.06).

Based on these two studies, it is possible to subtype meningiomas on the basis of advanced MRI sequences, i.e. diffusion weighted imaging and/or perfusion weighted imaging. However rCBV value could be better than ADC value to subtype meningiomas since P value for rCBV (0.02) (the current study) was well below the P value for ADC (0.06).

The current study had some limitations. The sample size of 35 was quite small. The rCBV or ADC maps were not used to target biopsies, which could have incurred sampling error due to tumor heterogeneity. Further studies with larger samples should be conducted to validate the results. More studies should also be conducted to subtype meningiomas on the basis of rCBV values in larger cohort of patients.

Presence of atypical and misleading features is not uncommon in meningiomas. There may be multiple histological variants and even a histologically typical meningioma may have misleading radiologic features not necessarily suggestive of meningiomas (Buetow et al., 1991). Histopathology cannot always be considered as a gold standard to grade tumors due to its own limitations as the malignant portions may be missed due to the limited tissue samples obtained after biopsy.

In case a very small portion of the representative area of tumor is included in the sample, which cannot be assessed by pathology, molecular genomic and quantitative proteomic techniques can be applied. Expression signatures in the form of amplification or metabolic signals can be picked up indicating the amplification of relevant genes or expression of putative proteins and then pathologists can even detect changes in that area in situ.

Using high density oligonucleotide microarray, Watson et al. (2002) reported successful grading of WHO grades I, II, and III meningiomas. Carvalho et al. (2007), using a combination of gene expression microarray and array Comparative Genomic Hybridization (aCGH), reported that meningiomas could be either low or high proliferative. However, the results could not suggest any definitive features to make precise molecular distinction of atypical meningiomas. In conjunction with Copy Number Association markers, the method can be used for histopathological grading to determine prognosis in cases of atypical meningiomas.

Molneon et al. (2010) using fluorescent in situ hybridization and high resolution magic angle spinning spectroscopy, identified distinct metabolic phenotypes for otherwise benign meningiomas. Multivariate analysis showed that benign meningiomas with complex karyo-type were metabolically closer to atypical meningiomas than other benign meningiomas. Meningiomas with chromosomal instabilities had more aggressive biochemistries regardless of their histological grade. More precise and rapid diagnosis and grading of meningioma can be done by combined and simultaneous measurement of metabolic, histopathology and molecular phenotype.

Sharma, Ray, Moiyadi, Sridhar and Srivastava (2014) through in silico quantitative functional analysis showed the modulation of different vital physiological pathways and provided grade specific protein signatures for meningiomas. To have a reliable meningioma grading system free of pitfalls, delineation of tumor boundaries is important. To avoid false negatives from molecular analyses experimenter needs to assure that the predictive marker molecules in reference are coming from the tumor tissue and not the surrounding normal tissue. Desorption electrospray ionization mass spectroscopy may be helpful to assess surgical and molecular margins between healthy and cancerous tissue in real time.

Brigham and Women’s Hospital (BWH) affiliated to Harvard Medical School (Boston, USA) could demonstrate remarkable differences in lipid profiles between cancerous and noncancerous tissue on intraoperative molecular characterization of brain tumors. Integration of this technique with stereotactic MS imaging platform and diagnostic exome sequencing may provide insights to develop a modality capable of precise characterization and grading of meningiomas.

Tumor cellularity can be imaged using functional Diffusion Maps (fDMs), which may act as surrogate brain imaging biomarkers. Computationally obtained fMDs can be used to establish correlation between water diffusivity and cellularity (Moffat et al., 2006). Such computations can be helpful to precisely predict the efficacy of tumor treatment. Molecular markers of hypoxia and vascularity correlate with dynamic contrast enhanced MRI in specific areas of intratumoral microenvironment and can well predict the patient’s outcome (Jensen & Lee, 2012; Jensen et al., 2014).

Advanced imaging modalities such as quantitative imaging biomarkers coupled with optical molecular imaging and nanotechnology can be useful tools to study tissue architecture from angiogenesis, vascularity, and permeability point of view to better address the grading and prognosis of meningiomas (Kircher et al., 2012; Taghva, Khalessi, Kim, Liu, & Apuzzo, 2010). However, in the settings where no sophistication is possible in radiological procedures, but modest molecular biological setup is available, simple molecular markers of diagnosis and prognosis can be applied.

Evidence from the literature suggests that MR imaging of cellularity and invasiveness, angiogenesis, capillary permeability, and microvasculature in meningioma can be corroborated using molecular markers such as Hypoxia-inducible factor-1α, matrix metalloproteinase-9, and vascular endothelial growth factor (Eberlin et al., 2013; Iwado et al., 2012; Jensen, Soleau, Bhayani, & Christiansen, 2002; Kaynars et al., 2008; Toh et al., 2014). Ongoing work in our laboratories is focused on finding a meaningful corroboration of molecular, imaging, and histopathological correlates as an aid to classify and grade meningiomas and predict the prognosis.

Perfusion parameters such as rCBV and percentage drop in peritumoral edema are useful to grade meningiomas. The rCBV values within the tumor may help to type, but not grade the meningiomas. Advancing knowledge of molecular pathological landscape of brain tumors and innovations in molecular genomic, spectroscopic, and proteomic technologies present a promising future for precise characterization and grading of meningiomas including atypical ones.

## Ethical Considerations

### Compliance with ethical guidelines

Ethical standards of the Institution Research Committee and 1964 Helsinki Declaration and its latest revision or comparable ethical standards were applied to the current study. All the study subjects signed informed consent forms.
